# Development and Evaluation of eHealth Services Regarding Accessibility: Scoping Literature Review

**DOI:** 10.2196/45118

**Published:** 2023-08-17

**Authors:** Marika Jonsson, Stefan Johansson, Dena Hussain, Jan Gulliksen, Catharina Gustavsson

**Affiliations:** 1 School of Electrical Engineering and Computer Science KTH Royal Institute of Technology Stockholm Sweden; 2 Habilitation & Health Region Västra Götaland Skövde Sweden; 3 Faculty of Engineering LTH Department of Design Sciences Certec Lund Sweden; 4 Center for Clinical Research Dalarna Uppsala University Falun Sweden; 5 School of Health and Welfare Dalarna University Falun Sweden; 6 Department of Public Health and Caring Sciences Uppsala University Uppsala Sweden

**Keywords:** accessibility, digital inclusion, disability, eHealth, Web Content Accessibility Guidelines, scoping literature review, universal design

## Abstract

**Background:**

Accessibility is acknowledged as a key to inclusion in the Convention of Rights for People with Disabilities. An inaccessible design can result in exclusion from eHealth and cause disability among people who have impairments.

**Objective:**

This scoping literature review aimed to investigate how eHealth services have been developed and evaluated regarding accessibility for people with impairments.

**Methods:**

In line with Arksey and O’Malley’s framework for scoping studies and using the Preferred Reporting Items for Systematic Reviews and Meta-Analyses extension for Scoping Reviews (PRISMA-ScR), we conducted a search in 4 databases (PubMed, Scopus, IEEE, and Web of Science) in October 2020 and an update of the search in June 2022. The search strategy was structured according to the PICO model as follows: Population/Problem, digital accessibility for users with impairment; Intervention, health care delivered by any digital solution; Comparison, not applicable; Outcome, use of and adherence to (1) Web Content Accessibility Guidelines (WCAG), (2) other accessibility guidelines, and (3) other means, for designing or evaluating accessibility in eHealth services. A Boolean search was conducted by combining terms related to accessibility and eHealth. All authors participated in screening abstracts according to the eligibility criteria. Each publication, containing a potentially relevant abstract, was read (full text) and assessed for eligibility by 2 authors independently and pairwise. Publications deemed eligible were read by all authors and discussed for consensus.

**Results:**

A total of 8643 publications were identified. After abstract screening, 131 publications remained for full-text reading. Of those, 116 publications were excluded as they did not meet the eligibility criteria. Fifteen publications involving studies of 12 eHealth services were included in the study. Of the 15 publications, 2 provided a definition of accessibility, 5 provided an explanation of accessibility, and 8 did not provide any explanation. Five publications used the WCAG to evaluate accessibility when developing eHealth services. One publication used International Organization for Standardization (ISO) 29138, ISO 2941, and ISO/International Electrotechnical Commission (IEC) 30071-1 standards together with the Spanish Association for Standardization (UNE) 139803 standard. Eleven publications used other means to address accessibility, including text-level grading; literature review about accessibility; user tests, focus groups, interviews, and design workshops with target groups of patients, relatives, and health care professionals; and comparative analysis of existing technical solutions to provide information about useful requirements.

**Conclusions:**

Although a clear definition of accessibility can enhance operationalization and thus measurability when evaluating accessibility in eHealth services, accessibility was insufficiently defined in most of the included studies. Further, accessibility guidelines and standards were used to a very limited extent in the development and evaluation of eHealth services. Guidelines for developing complex interventions that include guidance for accessibility are motivated to ensure that accessibility will be considered systematically in eHealth services.

## Introduction

Accessibility is acknowledged as a key to inclusion in the Convention of Rights for People with Disabilities [1]. Accessibility is defined as follows: “Extent to which products, systems, services, environments, and facilities can be used by people from a population with the widest range of characteristics and capabilities to achieve a specified goal in a specified context of use” [2].

The World Wide Web Consortium (W3C) published the first version of the Web Content Accessibility Guidelines (WCAG) in 1999 as a recommendation to provide accessible websites [3]. The World Health Organization recognizes that digitalization of health care can facilitate universal health coverage and points out that eHealth should be developed with accessibility in mind and designed to propel inclusiveness [4].

In 2016, the European Union Web Accessibility Directive mandated all European Union member states to introduce legislation on web accessibility in national legislation. The legislation regulates member states to comply with the European Standards (EN) 301549 standard [5], which builds on the technical standard WCAG 2.1 AA [6]. Similar legislation can be found in other parts of the world, for example, United States [7], New Zeeland [8], and Australia [9]. There is a large body of knowledge showing that web pages and web services most often do not comply with WCAG [10-14]. People with impairments report difficulties in using general functions on the internet, such as navigation, passwords, and services, compared with the general population [15]. The term impairment relates to problems in body function or structure, such as a significant deviation or loss [16]. Studies on accessibility in eHealth services have shown that the services have accessibility errors [17-19]. Inaccessible design can create exclusion from eHealth and cause disability among people with impairments [15].

The importance of accessible eHealth services has become particularly evident during the COVID-19 pandemic, and it has been suggested that eHealth contributes to reducing the spread of COVID-19 [20]. eHealth is defined as the use of information and communication technologies to exchange information digitally between a health care provider and a patient in order to achieve and maintain health [21]. The technical solutions that provide communication between a patient and a health care provider are often complex systems affected by legislation and regulations on issues, such as privacy, security, identification, internet-related infrastructure, interoperability, information sharing, accuracy, and accessibility. Despite standards and guidelines to improve digital accessibility and legislation to regulate the use of these guidelines, eHealth services are still perceived as inaccessible by users [15,22]. Therefore, it is important to investigate how research on eHealth services has included digital accessibility in the development and evaluation of eHealth services.

A scoping literature review is suitable to summarize findings in cases where the body of knowledge is heterogeneous in methods or disciplines [23]. A scoping review is also valuable to determine the extent to which and the way in which research has been conducted [24,25]. A framework for scoping reviews, developed by Arksey and O’Malley [26], suggests that scoping reviews can include relevant literature regardless of study design. Thus, a scoping literature review was considered suitable for this study since research on eHealth is conducted in several disciplines using different methods. Moreover, a scoping review was considered suitable to map how research is performed around the key concept of accessibility regardless of study design.

The aim of this scoping literature review was to investigate how eHealth services have been developed and evaluated regarding accessibility for people with impairments.

## Methods

### Search Strategy and Inclusion Criteria

The design of this scoping review was in line with Arksey and O’Malley’s framework for scoping studies following the 5-stage process: (1) identifying the research question; (2) identifying relevant studies; (3) study selection; (4) charting data; and (5) collating, summarizing, and reporting results [26]. The Preferred Reporting Items for Systematic Reviews and Meta-Analyses extension for Scoping Reviews (PRISMA-ScR) checklist and explanations [23] were used in the process (Multimedia Appendix 1). An a priori review protocol for the research group with inclusion and exclusion criteria was generated in line with the framework.

The research questions were as follows:

- How is digital accessibility addressed when eHealth services are developed and evaluated?
- Are the WCAG, other accessibility guidelines, or other means used to address digital accessibility in the development and evaluation of eHealth services?

#### Inclusion Criteria

Eligible publications were scientific peer-reviewed journal articles and conference papers published at any time and written in English or Swedish. The inclusion criteria for the search strategy were structured according to the PICO model [27]. PICO is an acronym for the 4 elements of Population/Problem, Intervention, Comparison, and Outcome that need to be described to formulate the research question and set the inclusion criteria. The criteria were as follows:

- Population/Problem: Digital accessibility for users with impairment
- Intervention: Health care delivered by any digital solution
- Comparison: Not applicable
- Outcome: Use of and adherence to (1) WCAG (any version), (2) other accessibility guidelines, and (3) other means, for designing or evaluating accessibility in eHealth services

#### Exclusion Criteria

Digital solutions that only monitored health without providing for health care and only monitored self-care were excluded, since the aim was to investigate complex systems with interaction or information exchange between patients and health care. For the same reason, websites that provided health information to the public were excluded. Literature written in languages other than English or Swedish was excluded. Literature reviews were excluded to avoid multiple data reporting.

### Information Sources, Literature Screening, and Selection

Since eHealth includes both medical science and information technology, databases from both areas were chosen. The research strategy was planned by the first (MJ), second (SJ), and last (CG) authors. Librarians were consulted in the choice of databases. There is a lack of consensus regarding the terms used for digital accessibility [28]. Several different terms and spellings are used regarding eHealth, and the search strategy included different spellings. Universal design and accessibility are broad terms also used in the context of the physical environment. A Boolean search was constructed with the decided MeSH (Medical Subject Headings) terms and other terms related to eHealth and terms related to accessibility. When the number of hits exceeded 5000, the hits were combined with other terms to narrow the result. Table 1 describes the search terms and search process exemplified with the search in PubMed. Search queries for Scopus, IEEE, and Web of Science are presented in Multimedia Appendix 2.

**Table 1:** The full electronic search strategy exemplified by the search in PubMed.

Search number	Search query	Number of hits	Specification of not included^a^ or included^b^
1	((((((ehealth) OR (e-health)) OR (e health)) OR (mhealth)) OR (mobile health)) OR (telemedicine)) OR (telerehabilitation)	74,326	Not included
2	(((((web accessibility) OR (digital accessibility)) OR (universal design)) OR (wcag)) OR (WCAG)) OR (accessibility guidelines)	54,890	Not included
3	(((((((ehealth) OR (e-health)) OR (e health)) OR (mhealth)) OR (mobile health)) OR (telemedicine)) OR (telerehabilitation)) AND (accessibility guidelines)	776	Included
4	(((((((ehealth) OR (e-health)) OR (e health)) OR (mhealth)) OR (mobile health)) OR (telemedicine)) OR (telerehabilitation)) AND (digital accessibility)	1666	Included
5	(((((((ehealth) OR (e-health)) OR (e health)) OR (mhealth)) OR (mobile health)) OR (telemedicine)) OR (telerehabilitation)) AND (web accessibility)	1692	Included
6	(((((((ehealth) OR (e-health)) OR (e health)) OR (mhealth)) OR (mobile health)) OR (telemedicine)) OR (telerehabilitation)) AND (wcag)	21	Included
7	(((((((ehealth) OR (e-health)) OR (e health)) OR (mhealth)) OR (mobile health)) OR (telemedicine)) OR (telerehabilitation)) AND (universal design)	113	Included

^a^Searches with >5000 hits were considered not included.

^b^A total of 4268 hits were included (searches 3-7); a total of 3285 hits were finally included (searches 3-7) after removing 983 duplicates.

The PubMed, Scopus, IEEE, and Web of Science databases were used. The database search was conducted by the first author in October 2020. The search result was exported to the Endnote reference program and duplicates were removed. Duplicates not detected by the “find duplicates function” were manually removed. Included references were exported to Rayyan, a collaborative online system for literature reviews [29]. Rayyan allows blinded individual decision-making, which can be unblinded for consensus discussions in the research group. All authors participated in reviewing the search results. Altogether, the research group represents experience from the areas of medical science, human-computer interaction, accessibility, and computer science. The review process started with establishing common ground for individual decision-making through an iterative process with a small number of publications using blinded reviews, and then discussing differences in assessment within the whole group. This step was iterated 3 times. Then, every publication was assessed for the eligibility criteria based on the titles and abstracts by 2 authors (MJ, SJ, DH, JG, and CG) independently. Each pair of authors resolved differences in assessment by consensus. Moreover, full-text reading and assessment of the eligibility criteria were performed by 2 authors (MJ, SJ, DH, JG, and CG) independently and then discussed for consensus. Publications with any disagreement in the pairs were assessed by the whole research group. An update of the search was conducted by the first author in June 2022, repeating the search process for publications published from year 2021 to 2022 (IEEE and Scopus) or between October 31, 2020, and June 20, 2022 (Web of Science and PubMed). After duplicates were removed from the second search, the publications were assessed for eligibility by the authors using the same procedure as the first search.

A data charting form was developed by the first (MJ) and last (CG) authors and discussed with the second (SJ) and fourth (JG) authors. The first author was responsible for extracting data from the included publications, and the first and last authors discussed and updated the data chart in an iterative process. The extracted data consisted of article characteristics (author, year, country, characteristics of the study population, and description of the eHealth service) and summarized data regarding the 2 research questions. The first author interpreted the meaning of the term “accessibility” by looking for a definition or explanation of accessibility. When accessibility was mentioned without definition or explanation, the first author interpreted the meaning of the term from the context. The extracted data were verified by all authors.

## Results

**Figure 1:**
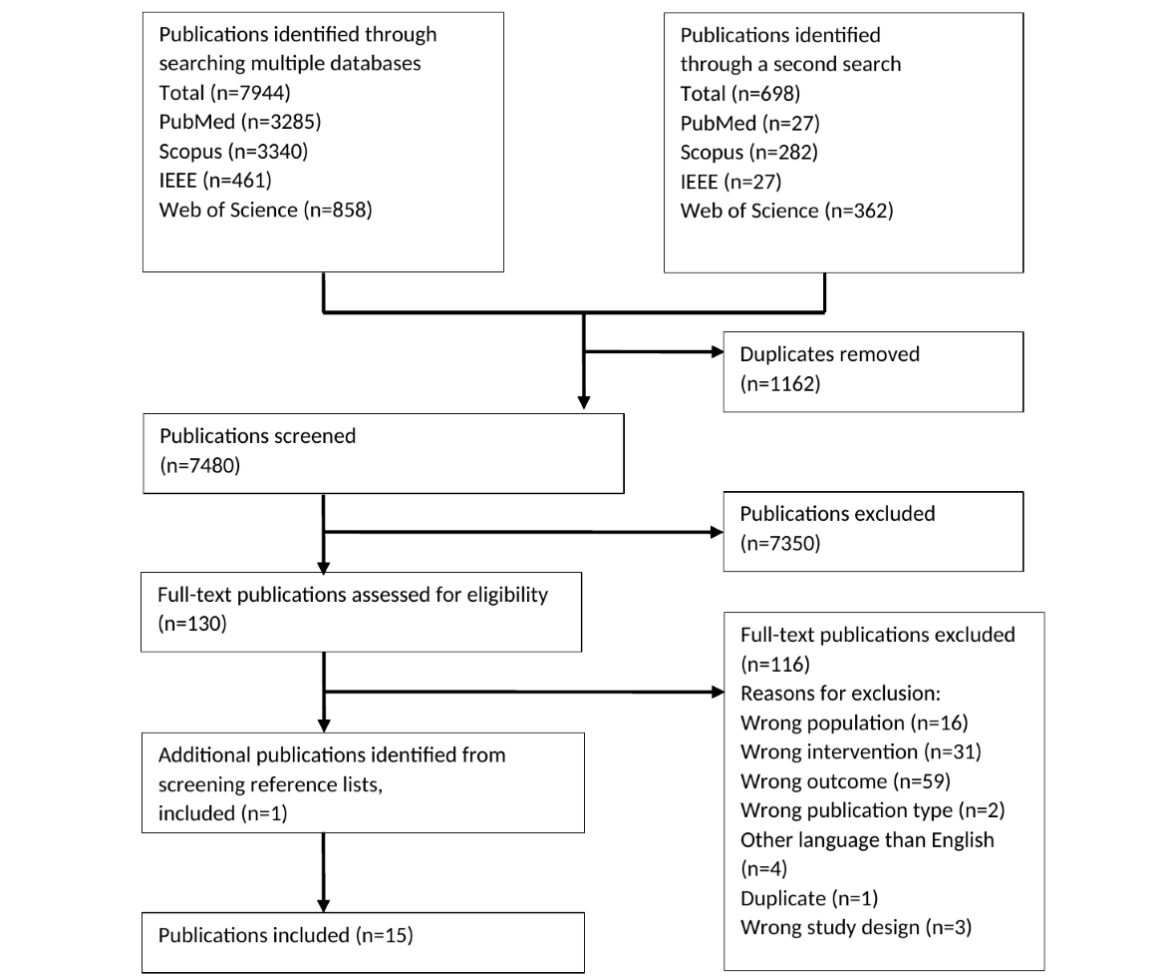
Flow diagram of the literature screening process.

After duplicates were removed, a total of 6911 publications were identified in the first search. The second search resulted in 569 publications. One additional publication was found through a search in one publication’s reference list and was included. A total of 131 publications remained for full-text assessment. Of those, 116 publications were excluded since they did not meet the eligibility criteria for the following reasons: (1) wrong population (ie, patient perspective was missing; n=16); (2) wrong intervention (ie, eHealth service did not include exchange of information or interaction between a patient and a health care provider, included self-care only, or a health care provider was missing; n=31); (3) wrong outcome (ie, accessibility was not addressed and no accessibility guidelines or other means were used; n=59); (4) wrong publication type (ie, not a scientific publication; n=2); (5) wrong language (ie, languages other than English or Swedish; n=4); (6) wrong study design (n=3); and (7) duplicate (n=1). Fifteen publications involving studies of 12 eHealth services were included in the study. Figure 1 provides a flow diagram of the screening process.

Table 2 provides information on the year of publication, place of origin, target population, target eHealth service, study design, methods, and main findings related to the research questions of the included publications. The publications were published from the year 2013 to 2022. All studies were conducted in Global North countries, except for 1 in Ecuador [30-32] and 1 in Indonesia [33]. The eHealth services in the included studies targeted different types of patients or diagnoses: hip arthroplasty surgery [30-32], chronic kidney disease [34], depression [35,36], intellectual disability [37,38], dexterity impairments [38,39], older adults with functional limitations [33,40], acquired brain injury [41], multiple sclerosis [42], children with long-term illness [43], and heart failure and chronic obstructive pulmonary disease [44]. The eHealth interventions in the studies consisted of rehabilitation after surgery [30-32], self-monitoring at home [34,40], mental health programs [35,36], supporting alternative communication [37], symptom reporting [43], facilitating appointments [33,38,41,44], self-management regimens [38,39,41,44], and a precision medicine tool [42].

In 3 publications, authors defined accessibility as how people can access and use systems regardless of abilities [30,31,35]. In 1 study, accessibility was defined as when a system is able to adapt itself to the preferences and characteristics of the user [34]. Two of the studies addressed accessibility as the usability of a product, service, environment, or facility by people with the widest range of capabilities as defined by the International Organization for Standardization (ISO) [32], and as universal design and user friendliness by referring to WCAG 2.0 [36]. One of the publications explained accessibility as when a goal can be easily achieved or accessed according to the person’s needs and the ease of using the service in a safe, comfortable, and independent way [33]. Eight studies did not provide a definition or explanation of accessibility. In those studies, the term accessibility was used in relation to the following: ease of use [38,42]; consistent design and instructive guidance explaining that cognitive, motivational, physical ability, and perception barriers influence usability [44]; language as an accessibility barrier [37]; simplicity of app design [41]; and mention as an aspect of familiarity of the terminology in technology [40]. The term accessibility was also used to indicate gaining access to something (ie, health care, login, and devices) [34,37,41,42].

Studies of three eHealth services, presented in 5 publications, used the WCAG to evaluate accessibility when developing eHealth services [30-33,36]. One publication used Web Accessibility Initiative (WAI) [30] and another used Web Accessibility Initiative: Ageing Education and Harmonization (WAI-AGE) [32] to guide the development and evaluation. One of the publications [34] used the Spanish Association for Standardization (UNE) 139803 standard, which is inspired by WCAG 2.0 together with the ISO 29138, ISO 9241, and ISO/International Electrotechnical Commission (IEC) 30071-1 standards.

Eight studies used other means than formal accessibility guidelines and standards to inform about or evaluate accessibility when developing eHealth services. These other means were as follows: text-level grading [35], literature review to inform about accessible text [40], user testing with a target group [33-35,38-40,44], user-centered design workshops with a target group of patients [37] or health professionals [43], focus groups with health professionals to gather meaningful requirements [43], focus groups with a target group of patients and their relatives to gather opinions on app content and design [41], interviews with a target group of patients regarding opinions and preferences on how and what to communicate within the eHealth service [43], a comparative analysis of existing technical solutions to inform about useful requirements [42], and the MOLD-US framework to assess usability issues [44].

**Table 2:** Summary of the characteristics of the included publications and results relating to the review questions.

Author, year, country	Development/evaluation	Study population	eHealth service	How was digital accessibility addressed?	What accessibility guidelines or other means were used?
Acosta-Vargas et al, 2018, Ecuador [31]	Evaluation	Elderly hip arthroplasty surgery patients	Telerehabilitation prototype platform	Study design: Descriptive Study procedures: Manual and computer-assisted evaluation of accessibility on random sample screens from the prototype platform, based on Website Accessibility Conformance Evaluation Methodology (WCAG-EM). Definition of accessibility: The level to which people can access websites, tools, and technologies regardless of technical, physical, or cognitive abilities. Measures: Accessibility operationalized as violation of the success criteria according to Web Content Accessibility Guidelines (WCAG) 2.0 measured by the web accessibility evaluation tools OpenWAX, Siteimprove Accessibility Checker, Tenon Check, and WAVE. Data analysis: Assessment of violation of the WCAG 2.0 success criteria and comparison between the tools for differences in violation of the success criteria.	Guidelines and standards: WCAG 2.0
Acosta-Vargas et al, 2019, Ecuador [30]	Evaluation	Elderly hip arthroplasty surgery patients	Telerehabilitation prototype platform	Study design: Descriptive Study procedures: Manual inspection of accessibility in 5 random samples of videos according to WCAG 2.0 and review of accessibility according to Web Accessibility Initiative (WAI) guidelines. The Photosensitive Epilepsy Analysis Tool (PEAT) was used for assessing sequences of luminance flashes and red flashes. Definition of accessibility: Universal accessibility is addressed in terms of the degree to which all people can use an object, visit a place, or access a service, regardless of their technical, cognitive, or physical capabilities. Measures: Accessibility operationalized according to WCAG 2.0. The PEAT measured flashing lights in videos. Data analysis: Descriptive statistical analysis of compliance with WCAG 2.0.	Guidelines and standards: WAI guidelines WCAG 2.0
Calle-Jimenez et al, 2019, Ecuador [32]	Development and evaluation	Elderly hip arthroplasty surgery patients	Telerehabilitation platform for postsurgical rehabilitation	Study design: Descriptive Study procedures: A selection of accessibility requirements based on WCAG 2.0 and WAI: Ageing Education and Harmonization (WAI-AGE) were assessed by web accessibility evaluation tools and complemented by manual inspection in a sample of web pages of the telerehabilitation platform. The web pages were then improved and retested. Definition of accessibility: The usability of a product, service, environment, or facility by people with the widest range of capabilities, as defined by the International Organization for Standardization (ISO). Measures: Accessibility operationalized according to WCAG 2.0 success criteria measured by the use of the web accessibility evaluation tools AChecker, TAW, and WAVE. Data analysis: Assessment of WCAG 2.0 success criteria and comparison between the tools for differences in the success criteria. Comparison of WCAG 2.0 success criteria in the telerehabilitation platform before and after accessibility improvements.	Guidelines and standards: WCAG 2.0 WAI-AGE
Calvillo-Arbizu, 2019, Spain [34]	Development and evaluation	Patients with chronic kidney disease	eHealth app (AppNephro) for self-monitoring at home	Study design: Descriptive and experimental Study procedures: Development of an eHealth app by (1) extracting design requirements from accessibility guidelines and standards, (2) performing interviews with nephrologists to analyze user accessibility needs in context, and (3) administering questionnaires to patients and caregivers to understand their experiences. Four patients performed accessibility and usability tests iteratively during the design and implementation of the app. Definition of accessibility: When a system can adapt itself to the needs, usage, and preferences of each user irrespective of the characteristics of the user. Measures: Patients’ needs and requirements of functions to be included in the app. Perceived accessibility, usability, and acceptability of the app. Data analysis: Method for analysis of interviews and questionnaires not reported. Proportion of participants (n=4) reporting that the app was easy to use and accessible for people with impairments.	Guidelines and standards: ISO 29138 ISO 9241 ISO/IEC 30071-1 Spanish standard UNE 139803 (inspired by WCAG 2.0) Other means
Clunne et al, 2018, Australia [35]	Evaluation	Post stroke aphasia patients with depression	Eight mental eHealth programs delivered through a website	Study design: Descriptive study of 8 eHealth programs and experimental study of Moodgym. Study procedures: Evaluation of general features and aphasia-specific communicative accessibility in 8 mental eHealth programs. One program, Moodgym, was selected for testing by 3 participants with poststroke aphasia. Participant performance (level of independent use) was evaluated by observation. A self-assessment questionnaire was used for participant’s satisfaction with Moodgym. Definition of accessibility: The ease by which patients can use the health care service in proportion to their needs, as well as the usability of the actual technology. Measures: Communicative accessibility operationalized as vocabulary and syntax, screen clarity, formatting, graphics, navigation, interface design, and media type. Text-level grading (by Flesch-Kincaid) as a part of aphasia-specific communicative accessibility evaluation. Accessibility measured by the observer assessing the user’s independent performance, and participant’s self-assessed satisfaction survey. Data analysis: Quantitative scoring of aphasia-specific communicative accessibility. Observer-rated participant level of independent use of Moodgym.	Other means
Gibson et al, 2020, United Kingdom [37]	Development and evaluation	People with mild intellectual disability (ID)	Augmented and alternative communication tools used in contact with primary health care	Study design: Descriptive Study procedures: User-centered design workshops and focus groups were conducted with participants with ID, focusing on barriers to accessing health care, evaluating suitable medical images, creating paper prototypes, identifying design requirements, and giving feedback on digital prototypes. Accessible language guidelines (not further specified) were used. Definition of accessibility: Not reported. Accessibility was mentioned in relation to access to health care, language as an accessibility barrier, and explanation that a single picture style could lead to accessibility issues. Measures: Participants’ opinions about barriers to access and requirements for design. Data analysis: Thematic analysis of transcriptions of audio-recorded sessions.	Other means
Kascak et al, 2013, United States [40]	Development and evaluation	Older adults with functional limitations relating to age	Remote patient monitoring (RPM) devices, and case study on the BL Healthcare Access Tablet	Study design: Experimental Study procedures: A literature review was conducted to gather design criteria for RPM. User tests by 6 volunteering older adults in the BL Healthcare Access Tablet and in mock-ups. Redesign of RPM with improvements and repeated usability tests by 5 of the 6 volunteers. Definition of accessibility: Not reported. Accessibility was mentioned in relation to access to health care and making data accessible for health care providers, and with reference to a paper addressing accessibility issues for elders. Measures: User behavior, experience, and opinion of usability of the BL Healthcare Access Tablet (eg, easy to navigate the user interface and functionality). Data analysis: Number of participants reporting aspects of usability as “excellent,” “good,” or “neutral.”	Other means
Osborne et al, 2020, United States [41]	Development	Persons with acquired brain injury (ABI)	Mobile health app	Study design: Descriptive Study procedures: Focus groups with guided discussions with people with ABI, their care partners, and therapists to identify meaningful content and features for user-centered app design and digital accessibility. Definition of accessibility: Not reported. Accessibility was mentioned in relation to accessibility to health care, simplicity of app design, and app design for visual impairment. Measures: ABI participants’ experiences with mobile use before and after brain injury, and opinions on app content and design. Professionals’ opinions on app content and design, and facilitators and barriers to mHealth intervention from the clinical perspective. Access to health care, login, sharing information, and digital accessibility regarding font size, colors, pictures instead of words, and auditory components. Data analysis: Thematic analysis of transcribed audio recordings.	Other means
Schleimer et al, 2020, United States [42]	Development	Adult patients with multiple sclerosis (MS)	Open MS BioScreen	Study design: Descriptive Study procedures: Human-centered design and development approach to create the Open MS BioScreen platform. Interviews with patients with MS, health care professionals working with MS, advocacy representatives, and industry representatives, and observations of 5 health care professionals at work were conducted to gather insights of patients’ needs. A comparative analysis of existing patient-facing tools was conducted to define key features. Then, a mock-up was created and shown to health care professionals, patients with MS, and MS support groups for feedback. A web-based prototype was built and tested by people with MS, and discussed in interviews with patients, health care professionals, advocacy representatives, and industry representatives. Definition of accessibility: Not reported. The term was mentioned in relation to a freely available platform and “easy to use” (eg, web-based translation into other languages and possibility to request assistance from a proxy user). Measures: Perceived needs of MS patients in an eHealth service, and perceived usability and accessibility of the prototype. Data analysis: Comparative analysis of available technical solutions. Interviews analyzed with grounded theory. Comparison of patient-entered and study clinician–entered clinical data expressed with the percentage of concordance.	Other means
Wildenbos et al, 2019, Netherlands [44]	Evaluation	Patients with heart failure or chronic obstructive pulmonary disease (COPD)	App 1 facilitating hospital appointment attendance and App 2 for self-monitoring	Study design: Descriptive case study Study procedures: Usability issues in 2 mHealth apps were collected by user tests performed by 3 patients with heart failure or COPD using a “think aloud” protocol. Test sessions were video recorded and field notes were taken. Definition of accessibility: Not reported. Usability was addressed; cognitive, motivational, physical ability, and perception barriers were explained as influencing usability. Measures: Participants’ task performance and opinions of using the apps. Severity of usability issues and category of usability issues: motivation, cognition, perception, and physical ability. Data analysis: Completion rate and time for the performed tasks presented as the proportion (%) of completed tasks and average time of performing the tasks. The severity of usability issues was ranked with Nielsen classification. The type of usability issue was categorized with the MOLD-US framework.	Other means: MOLD-US framework
Yogarajah et al, 2020, Norway and Sweden [36]	Evaluation	Adults with major depression and stable medication	Five applications providing internet-delivered cognitive behavioral therapy (CBT): eMeistring, Assistert Selvhjelp, MoodGym (2.0), Psyktools, Internetpsykiatri	Study design: Descriptive Study procedures: Usability and accessibility in 6 internet-delivered CBT treatments were assessed by one of the researchers acting as a patient. Two test tools (W3 checker and Color Contrast Analyzer) were used to assess if the apps met the WCAG 2.0 success criteria. Definition of accessibility: Universal design and user friendliness according to WCAG 2.0. Measures: Accessibility operationalized as meeting the WCAG 2.0 success criteria. Data analysis: Conformance evaluations according to WCAG 2.0 for each application, and then, a summary on the level of conformance by reporting on the proportions of compliance divided according to the WCAG levels A, AA, and AAA.	WCAG 2.0
Yu et al, 2019, United States [38]	Development and evaluation	Users with intellectual disabilities or dexterity impairment	iMHere, support for self-management regimens	Study design: Experimental Study procedures: Usability testing of redesigned modules in iMHere in a 1-week field trial with 9 participants who had dexterity impairment. Five participants had cognitive impairment. After the field trial, laboratory setting user tests of task performance were undertaken with interviews and the think aloud method. User tests with evaluation of task performance (user effort, task completion, task time, and error rate) and participant ranking of the importance of usability and accessibility features. Definition of accessibility: Not reported. Authors declared focusing on accessibility redesign and focusing on physical presentation and navigability. Accessibility was mentioned in relation to the requirements of users with intellectual disability and dexterity impairments described as relating to easy to understand and use manually: accessibility can be improved by using simple and common words, using shortcuts for navigation, and hiding unused modules. Measures: Accessibility was operationalized as participants’ opinions on the importance of accessibility features and the evaluation of performance measured as user effort, task completion, task time, and error rate. Usability measured by the Telehealth Usability Questionnaire (TUQ). Data analysis: Method for analyzing interviews not reported. Statistical analyses of importance ranking, performance of user effort, task time, and error rate with the Shapiro-Wilk test. Overall average scores and individual factor scores were calculated for the TUQ.	Other means
Syahrina et al, 2021, Indonesia [33]	Evaluation	Older adults with functional limitations relating to age	Halodoc, eHealth app with several features. Evaluation on consultation with a doctor through chat, booking of appointments to the hospital, and purchasing of medicine. The target group includes all Indonesian people.	Study design: Experimental Study procedures: Computer-assisted evaluation of accessibility and user testing. Google Accessibility Scanner was used to detect accessibility errors regarding content labels, touch target size, clickable items, and text and image contrast. Semistructured interviews for participant background information. Video-recorded user testing with given tasks performed by 10 people older than 60 years. Reflective questions at the end of the session. Definition of accessibility: Accessibility was defined as: (1) when a goal can easily be achieved or accessed; (2) the ease of use of a service in a safe, comfortable, and independent way; (3) the facility provided that is useful in realizing equal opportunities. Measures: Accessibility was operationalized as the number of detected errors in the scanner test, and the task completion time, number of errors, and user experience in user testing. Data analysis: Scanner test: Number of errors. User testing: Average task completion time and number of errors; user experience expressed in follow-up questions categorized as overall impression, registration, and user interface–related problems.	Guidelines and standards: WCAG 2.0 Other means
Chowdhary et al, 2022, United States [39]	Development and evaluation	People with disabilities and dexterity impairments	iMHere 1.0 eHealth app, modules MyMeds and SkinCare	Study design: Experimental Study procedures: User’s dexterity impairment level assessed before task user testing and development of iMHere. Definition of accessibility: Not reported. Accessibility was mentioned in relation to consistent design, instructive guidance, simpler process, and 10 accessibility features. Measures: Accessibility was operationalized as 10 accessibility features: customized module list, text display, background and color, button size, keyboard, navigational shortcut, possibility to import photos of medication, color-coded features in a module, text, and audio guidance. Usability measured with the TUQ. User preference measured as the opinion of the preferred design, and choosing between the old and new design. Data analysis: Descriptive statistics on average task time, number of actions for each task, error rate, and level of independence in solving tasks. Comparison of TUQ mean scores of the original and redesigned modules. Participant ranking of the importance of accessibility features.	Other means
Wiljén et al, 2022, Sweden [43]	Development and evaluation	Children with long-term illness	eHealth app Pictorial Support in Person-Centered Care for Children called PicPecc. App for self-report and managing symptoms at a hospital or at home.	Study design: Descriptive Study procedures: User-centered design process with 2 phases. The principles of universal design were used to guide accessibility together with experts in universal design to facilitate accessibility for all users, regardless of ability and needs. Phase 1 contained 3 stages: analysis, design, and evaluation. Focus groups and interviews with children, parents, and health care professionals; workshops with experts and developers; and user experience workshops to develop a mock-up. Phase 2 contained 1 stage: evaluation. Focus groups and interviews with children, parents, and health care professionals involving a mock-up. Definition of accessibility: Not reported. Accessibility was explained as universal design to facilitate accessibility for users with all kinds of abilities and needs, and it can be provided using pictures, audio, and easy-to-read texts. Measures: Accessibility was operationalized as the participants’ shared experiences and opinions on the mock-up. Data analysis: Interpretive description of transcribed audio data. The software NVivo 12 Pro was used to find patterns and relationships among codes.	Other means

## Discussion

### Principal Results

In the studies included in this scoping literature review, digital accessibility in eHealth services was addressed in several different ways, mostly starting with the user’s abilities, and also considering the system’s potential to adapt to the user. Only 2 of the 15 publications used a formal definition of accessibility with references to the ISO standard or WCAG, and 6 of the 15 publications targeting 4 eHealth services used a web accessibility guideline or standard in the development or evaluation of eHealth services.

In many studies, the term usability was used as an equivalent to accessibility. For example, a publication [36] explained the WCAG accessibility guideline as a standard for evaluating the usability of software systems, which takes into account new technologies, different user agents, and universal design to a sufficient degree [36]. Awareness of the presence of specific and precise definitions of accessibility seems to be low in the field of eHealth development. It seems that the typical development process of eHealth starts from a usability perspective and then subsequently provides some extra attention to a very specific target group (ie, people with a specific diagnosis). To create a holistic view on accessibility, the W3C provides guidance on how to combine formal WCAG evaluation in collaboration with users having impairments [45]. When designing for a specific target population, it is important to recognize that people in the target population might also have a range of other issues and impairments that affect interaction with technology and impede accessibility. Therefore, a holistic view is vital, and we argue that it is important to always consider and comply with the WCAG during the development process.

Not taking a holistic approach is erroneous since even if the eHealth service as such targets people with a specific diagnosis, those people might also have a range of other issues and impairments when interacting with technology, which can impede accessibility.

A problem with the lack of consensus on the definition of accessibility is the risk of less accessibility for the target audience [28]. There are several ISO standards targeting accessibility issues, and the concept of accessibility is strongly related to the concept of usability, recognizing that accessibility contributes to achieving usability [46]. However, usability does not automatically cover accessibility. This suggests that it is important to use the formal predefined accessibility terms with reference to the WCAG or ISO standard to avoid confusion and to be clear which kind of accessibility is targeted.

Six publications targeting 4 eHealth services used a web accessibility guideline or standard in the development or evaluation of the eHealth services. It is reasonable to believe that researchers who use the WCAG will also use the term accessibility or WCAG as a keyword. Therefore, it is plausible that our findings reflect how infrequently accessibility guidelines have been used when developing or evaluating eHealth services. The low number indicates that eHealth services may exclude people with impairments. Previous research confirms that accessibility is insufficient in eHealth and argues that the WCAG standards are important but not sufficient to develop accessible eHealth services [47]. However, not using accessibility guidelines or standards and only relying on information from users can increase the risk of missing several aspects of accessibility. The information that users provide during user testing is based on their own experience. Therefore, it is most likely not based on any knowledge about accessibility requirements related to the quality of the code or the technical construction of the eHealth service that is tested. Previous research has shown that developers and user experience (UX) professionals have limited knowledge about accessibility [48,49], implying that relying on the knowledge of the developers and UX designers might increase the risk of missing accessibility aspects. Thus, to accurately address accessibility, it is favorable to combine information from accessibility guidelines, the experiences of users in the target group, and the knowledge of developers and UX designers.

Although the COVID-19 pandemic has drawn attention to the need of offering eHealth services to all people, we only found 3 studies [33,39,43] published after 2020 that addressed accessibility. In the framework for implementation research by Peters et al, it is recommended that the target population be described in sufficient detail [50]. When developing eHealth services, it is important to recognize that patients with a specific diagnosis can also have a range of other impairments. Thus, when describing the target population, it is important to also describe the heterogeneity within the population, addressing all kinds of impairments even though they may not be related to the diagnosis. Designing for the widest range of capabilities should be the goal. Complying with accessibility guidelines increases the inclusion of a wide range of user characteristics and reduces the risk of digital exclusion.

Several Global North countries [6-9] have regulated the level of compliance to the WCAG that public websites and applications must achieve, indicating that research addressing digital accessibility for both existing and new eHealth services will increase in this part of the world. It is notable that we only found 2 studies outside the Global North, although several other countries, for example, India and Taiwan, are listed as having mandatory policies referring to the WCAG [51]. The reasons the WCAG are not used more often in the development of eHealth services, despite being available for more than two decades [11], could be that the guidelines are not well known and that the guidelines and standards are difficult to use.

eHealth is developed within multiple disciplines and some of the disciplines may be less aware of digital accessibility. Medical researchers and developers of eHealth may rely on guidelines and information that do not cover digital accessibility. One example is the widely used Medical Research Council (MRC) guidelines for developing and evaluating complex interventions [52], which do not contain any information about accessibility in eHealth. An update of the MRC guidelines targeting eHealth, with special reference to relevant accessibility guidelines and standards, could strengthen future research and development of accessibility in eHealth interventions.

In what we categorized as “other means,” we found a wide range of methods not using accessibility standards or guidelines for evaluating accessibility in eHealth services. This indicates that either accessibility evaluation is not known or there is a lack of knowledge regarding the differences in usability and accessibility. In some of the studies, usability was used as an equivalent to accessibility, although usability does not automatically cover accessibility. For example, in a publication [35], it was explained that accessibility is the ease with which patients can use the health care service in proportion to their needs, as well as the usability of the actual technology.

Using other means to develop or evaluate eHealth may be a way of including the assessment of cognitive accessibility, which is not well covered in the WCAG. WCAG 2.1 and older versions have been criticized for not covering accessibility issues when users have cognitive disability [53,54]. Hence, to cover all relevant accessibility issues, it is suggested to combine the WCAG with other means. Those other means should be a combination of guidelines for cognitive accessibility, understandable text or content, and user testing with a diverse set of users with impairments.

**Figure 2:**
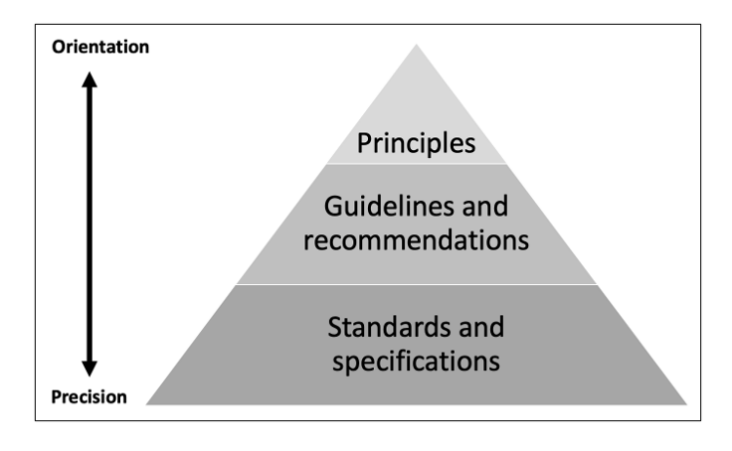
The relations among principles, guidelines, and standards, and guidance for precision and orientation toward accessibility.

In summary, the means used for dealing with accessibility in the included publications can be referred to principles (eg, the principles for universal design), guidelines (eg, some kind of heuristics), and standards (eg, WCAG 2.1 AA or HTML5) (Figure 2). Principles provide overall orientation and connect the development of the eHealth service to concepts of fairness, human rights, inclusion, and participation. Guidelines and recommendations are important to create awareness and point out a direction for accessibility in the service. Standards and specifications provide the level of precision needed to ensure actual accessibility in eHealth [55,56]. Most of the publications included in our study did not refer to standards or specifications. However, for many people with impairments, accessibility is in the details, meaning that a high level of precision and compliance to detailed specifications is important to ensure accessibility. For example, the technical construction of an eHealth service needs to be very precise to be interoperable with the assistive technology used by blind people or people who use navigation techniques other than moving a mouse pointer and clicking on objects. Most guidelines and principles do not provide sufficient precision.

If the development of an eHealth service solely relates to principles and guidelines, as for most of the publications included in this scoping review, the approach is still far better than not considering accessibility at all. However, those services will probably not conform to the legally mandated level of technical accessibility stated in the EN 310 549/WCAG 2.1 AA standard or in the ISO 21 801-1 standard for cognitive accessibility. Our findings show that although the publications included in this review often advocated for usability, accessibility was not addressed with the level of precision needed to ensure accessible eHealth. Even studies that claimed to have followed guidelines still only investigated usability measures, leaving accessibility as a neglected issue in studies developing eHealth services.

### Strengths and Limitations

A strength of this study is the construction of a thorough search strategy with a review protocol before conducting the literature search, which increased the reproducibility and reliability of the study [57]. The process of setting common ground for individual decision-making, the consensus discussions, and the approach of all researchers reading the included publications in full text strengthen the systematic and reproducible study selection [23]. Another strength of this study is that the research group represented experience from several important disciplines and competences with regard to the development of eHealth services: medical science, human-computer interaction, accessibility, and computer science. This increased the validity of the review process, as the publications assessed in this study came from several scientific disciplines and required in-depth knowledge in the aspects of accessibility in digital technology and health care delivery. These together increase the trustworthiness of the results. All but 2 of the studies in this literature review were conducted in the context of a Global North country. Thus, the findings may have limited generalizability to countries outside the Global North. It is possible that research has been published in languages other than English or Swedish, and thus, there may be studies in other languages that were not included in this study.

### Conclusions

Although a clear definition of accessibility can enhance operationalization and thus measurability when evaluating accessibility in eHealth services, the results of this literature review show that accessibility was insufficiently defined in most of the included studies. Further, the results show that accessibility guidelines and standards were used to a very limited extent in the development and evaluation of eHealth services. Guidelines for developing complex interventions that include guidance for accessibility are motivated to ensure that accessibility will be considered systematically in eHealth services.

## Appendix

Multimedia Appendix 1 PRISMA-ScR (Preferred Reporting Items for Systematic Reviews and Meta-Analyses extension for Scoping Reviews) checklist.

Multimedia Appendix 2 Final search strategy for PubMed, Scopus, IEEE, and Web of Science.

## Abbreviations

EN: European Standards
IEC: International Electrotechnical Commission
ISO: International Organization for Standardization
MRC: Medical Research Council
PICO: Population, Intervention, Comparison, and Outcome
PRISMA-ScR: Preferred Reporting Items for Systematic Reviews and Meta-Analyses extension for Scoping Reviews
UNE: Spanish Association for Standardization
UX: user experience
W3C: World Wide Web Consortium
WCAG: Web Content Accessibility Guidelines
