# Supplementary material for: Development and Evaluation of eHealth Services Regarding Accessibility: Scoping Literature Review
Source: J Med Internet Res. 2023 Aug 17;25:e45118. doi: 10.2196/45118 (PMC10472171; doi:10.2196/45118)
Supplement: Multimedia Appendix 2 [file jmir_v25i1e45118_app2.pdf]

# Appendix 2

## Final search strategy for PubMed, Scopus, IEEE, and Web of Science

### PubMed

The search was conducted with search queries.

| Search queries |
|---|
| |
| ((((((ehealth) OR (e-health)) OR (e health)) OR (mhealth)) OR (mobile health)) OR (telemedicine)) OR (telerehabilitation)) AND (accessibility guidelines) |
| ((((((ehealth) OR (e-health)) OR (e health)) OR (mhealth)) OR (mobile health)) OR (telemedicine)) OR (telerehabilitation)) AND (digital accessibility) |
| ((((((ehealth) OR (e-health)) OR (e health)) OR (mhealth)) OR (mobile health)) OR (telemedicine)) OR (telerehabilitation)) AND (web accessibility) |
| ((((((ehealth) OR (e-health)) OR (e health)) OR (mhealth)) OR (mobile health)) OR (telemedicine)) OR (telerehabilitation)) AND (wcag) |
| ((((((ehealth) OR (e-health)) OR (e health)) OR (mhealth)) OR (mobile health)) OR (telemedicine)) OR (telerehabilitation)) AND (universal design) |

### Scopus

The search was conducted with one search query that was refined with the function 'search within results'. Only the refined searches were included in the study.

| | Search strategy |
|---|---|
| | |
| **Search query** | |
| | (ehealth OR "e ehealth" OR e-health OR mhealth OR "mobile health" OR telemedicine OR telerehabilitation ) |
| | |
| **Refined with** | |
| | web accessibility |
| | digital accessibility |
| | universal design |
| | wcag |
| | WCAG |
| | accessibility guidelines |

### IEEE

The search was conducted with search queries.

| Search queries |
|---|
| |
| ((((((("All Metadata":ehealth) OR "All Metadata":e-health) OR "All Metadata":e health) OR "All Metadata":mhealth) OR "All Metadata":mobile health) OR "All Metadata":telemedicine) OR "All Metadata":telerehabilitation) AND "All Metadata":digital accessibility) |
| ((((((("All Metadata":ehealth) OR "All Metadata":e-health) OR "All Metadata":e health) OR "All Metadata":mhealth) OR "All Metadata":mobile health) OR "All Metadata":telemedicine) OR "All Metadata":telerehabilitation) AND "All Metadata":web accessibility) |

| |
|---|
| ((((((("All Metadata":ehealth) OR "All Metadata":e-health) OR "All Metadata":e health) OR "All Metadata":mhealth) OR "All Metadata":mobile health) OR "All Metadata":telemedicine) OR "All Metadata":telerehabilitation) AND "All Metadata":universal design) |
| ((((((("All Metadata":ehealth) OR "All Metadata":e-health) OR "All Metadata":e health) OR "All Metadata":mhealth) OR "All Metadata":mobile health) OR "All Metadata":telemedicine) OR "All Metadata":telerehabilitation) AND "All Metadata":WCAG) |
| ((((((("All Metadata":ehealth) OR "All Metadata":e-health) OR "All Metadata":e health) OR "All Metadata":mhealth) OR "All Metadata":mobile health) OR "All Metadata":telemedicine) OR "All Metadata":telerehabilitation) AND "All Metadata":accessibility guidelines) |

## Web of Science

The search was conducted with one search query that was refined with the function 'search within results'. Only the refined searches were included in the study.

| | Search strategy |
|---|---|
| | |
| **Search query** | |
| | TOPIC: (ehealth) OR TOPIC: (e-health) OR TOPIC: (e health) OR TOPIC: (mhealth) OR TOPIC: (mobile health) OR TOPIC: (telemedicine) OR TOPIC: (telerehabilitation) |
| | |
| **Refined with** | |
| | digital accessibility |
| | web accessibility |
| | wcag |
| | universal design |
| | accessibility guidelines |
| | |
